# Pentachlorophenol Degradation by *Janibacter* sp., a New Actinobacterium Isolated from Saline Sediment of Arid Land

**DOI:** 10.1155/2014/296472

**Published:** 2014-09-17

**Authors:** Amel Khessairi, Imene Fhoula, Atef Jaouani, Yousra Turki, Ameur Cherif, Abdellatif Boudabous, Abdennaceur Hassen, Hadda Ouzari

**Affiliations:** ^1^Université Tunis El Manar, Faculté des Sciences de Tunis (FST), LR03ES03 Laboratoire de Microorganisme et Biomolécules Actives, Campus Universitaire, 2092 Tunis, Tunisia; ^2^Laboratoire de Traitement et Recyclage des Eaux, Centre des Recherches et Technologie des Eaux (CERTE), Technopôle Borj-Cédria, B.P. 273, 8020 Soliman, Tunisia; ^3^Université de Manouba, Institut Supérieur de Biotechnologie de Sidi Thabet, LR11ES31 Laboratoire de Biotechnologie et Valorization des Bio-Geo Resources, Biotechpole de Sidi Thabet, 2020 Ariana, Tunisia

## Abstract

Many pentachlorophenol- (PCP-) contaminated environments are characterized by low or elevated temperatures, acidic or alkaline pH, and high salt concentrations. PCP-degrading microorganisms, adapted to grow and prosper in these environments, play an important role in the biological treatment of polluted extreme habitats. A PCP-degrading bacterium was isolated and characterized from arid and saline soil in southern Tunisia and was enriched in mineral salts medium supplemented with PCP as source of carbon and energy. Based on 16S rRNA coding gene sequence analysis, the strain FAS23 was identified as *Janibacter* sp. As revealed by high performance liquid chromatography (HPLC) analysis, FAS23 strain was found to be efficient for PCP removal in the presence of 1% of glucose. The conditions of growth and PCP removal by FAS23 strain were found to be optimal in neutral pH and at a temperature of 30°C. Moreover, this strain was found to be halotolerant at a range of 1–10% of NaCl and able to degrade PCP at a concentration up to 300 mg/L, while the addition of nonionic surfactant (Tween 80) enhanced the PCP removal capacity.

## 1. Introduction

The polyorganochlorophenolic (POP) compounds have been extensively used as wide spectrum biocides in industry and agriculture [1]. The toxicity of these compounds tends to increase according to their degree of chlorination [2]. Among chlorinated phenols, pentachlorophenol (PCP) has been widely used as wood and leather preservative, owing to its toxicity toward bacteria, mould, algae, and fungi [3]. However, PCP is also toxic to all forms of life since it is an inhibitor of oxidative phosphorylation [4]. The extensive exposure to PCP could cause cancer, acute pancreatitis, immunodeficiency, and neurological disorders [5]. Consequently, this compound is listed among the priority pollutants of the US Environmental Protection Agency [6]. Moreover, it is recalcitrant to degradation because of its stable aromatic ring and high chloride contents, thus persisting in the environment [7]. Although contamination of soils and waters with chemically synthesized PCP is a serious environmental problem, their remediation may be possible using physical, chemical, and biological methods [8]. Bioremediation represents a choice process, thanks to its low costs and reduction of toxic residue generated in the environment. The biodegradation of PCP has been studied in both aerobic and anaerobic systems. Aerobic degradation of PCP especially has been extensively studied and several bacterial isolates were found to degrade and use PCP as a sole source of carbon and energy. The most studied aerobic PCP-degrading microorganisms included* Mycobacterium chlorophenolicum* [9],* Alcaligenes* sp. [10],* Rhodococcus chlorophenolicus *[11],* Flavobacterium* [12],* Novosphingobium lentum* [13] and* Sphingomonas chlorophenolica* [14],* Bacillus* [15],* Pseudomonas* [16], and* Acinetobacter* [17], as well as some fungi species. Saline and arid environments are found in a wide variety of aquatic and terrestrial ecosystems. A low taxonomic biodiversity is observed in all these saline environments [18], most probably due to the high salt concentrations prevailing in these environments. Moreover, the biodegradation process is difficult to perform under saline conditions [19]. Besides these metabolical and physiological features, halophilic and halotolerant microorganisms are known to play important roles in transforming and degrading waste and organic pollutants in saline and arid environment [20]. These microorganisms, particularly actinobacteria, are frequently isolated from extreme environments such as Sabkha, Chott, and Sahara which are known to have a great metabolic diversity and biotechnological potential. The occurrence of actinobacteria in saline environment and their tolerance to high salt concentrations were thus described [21]. However, few actinobacteria genera, such as* Arthrobacter* [22] and* Kocuria* [23], were reported for PCP-degradation process. The genus* Janibacter* which is recognized by Martin et al. [24] belongs to the family Intrasporangiaceae in the Actinomycetales order and included five major species,* J. limosus* [24],* J. terrae* [25],* J. melonis* [26],* J. corallicola* [27], and* J. anophelis* [28]. Interestingly, most of these species were reported for their ability to degrade a large spectrum of aromatic and/or chlorinated compounds including polychlorinated biphenyls [29], monochlorinated dibenzo-*p*-dioxin [30], dibenzofuran [31], anthracene, phenanthrene [32], dibenzo-p-dioxin, carbazole, diphenyl ether, fluorene [33], and polycyclic aromatic hydrocarbons [34]. However, no data reporting PCP degradation by* Janibacter* members was described. PCP and other POP compounds shared many physical properties, which limited biodegradation processes, and one of these properties was their lower solubility and therefore low bioavailability to the degrading bacteria. Nevertheless, the use of surfactants such as Tween 80 has the potential to increase the biodegradation rates of hydrophobic organic compounds by increasing the total aqueous solubility of these pesticides [35]. In this study, we evaluated for the first time the PCP removal potential, under different physicochemical conditions, by* Janibacter* sp., a halotolerant actinobacterium member isolated from arid and saline land in southern Tunisia.

## 2. Materials and Methods

### 2.1. Chemicals and Solvents

PCP (MW 266.34 and >99% purity) and acetonitrile (HPLC grade) were purchased from Sigma Aldrich (USA). All other inorganic chemicals used to prepare the different media are commercially available with highest purity and are used without further purification.

### 2.2. Sample Collection and PCP-Degrading Bacterium Isolation

The sediment samples were collected in March 2011 from arid and saline ecosystems belonging to the site “Sebkha El Naouel” with GPS coordinates: N 34°26′951′′ E 09°54′102′′ altitude 150 ft/46 m, in southern Tunisia. Bacterial isolation was performed as described by Rösch et al. [36] with some modifications: 10 g of soil sample was suspended in 100 mL of phosphate-buffered salt solution (137 mM NaCl, 2.7 mM KCl, 10 mM Na_2_HPO_4_, and 2 mM KH_2_PO_4_) and stirred vigorously for 30 min. The soil suspension was diluted and 0.1 mL sample was spread on the surface of yeast extract-mannitol medium (YEM). YEM medium contained the following components at the specified concentrations (in g/L): mannitol, 5; yeast extract, 0.5; MgSO_4_ *·*7H_2_O, 0.2; NaCl, 0.1; K_2_HPO_4_, 0.5; Na gluconate, 5; agar, 15; pH = 6, 8. After sterilization for 20 min at 120°C, 1 mL of 16.6% CaCl_2_ solution was added to 1 liter of YEM medium (1 : 1000). The plates were then incubated at 30°C for 7 days. Pure cultures of the isolates were obtained by streaking a single colony on the same medium.

### 2.3. 16S rRNA Gene Amplification and Sequence Analysis

For DNA extraction, the FAS23 strain was grown in tryptic soy broth (TSB) containing (in g/L) casein peptone, 17; soya peptone, 3; glucose, 2.5; sodium chloride, 5; dipotassium hydrogen phosphate, 4. DNA extraction was performed using CTAB/NaCl method as described by Wilson [37] and modified by using lysozyme (1 mg/mL) for cell wall digestion. The 16S rRNA gene was amplified using universal primers SD-Bact-0008-a-S-20 (5′-AGA GTT TGA TCC TGG CTC AG-′3) and S-D-Bact-1495-a-A-20 (5′-CTA CGG CTA CCT TGT TAC GA-′3) [38]. PCR was performed in a final volume of 25 *μ*L containing 1 *μ*L of the template DNA; 0.5 *μ*M of each primer; 0.5 *μ*M of deoxynucleotide triphosphate (dNTP); 2.5 *μ*L 10X PCR buffer for Taq polymerase; MgCl_2_ 1.5 mM; 1 UI of Taq polymerase. The amplification cycle was as follows: denaturation step at 94°C for 3 min, followed by 35 cycles (45 sec at 94°C, 1 min at 55°C, and 2 min at 72°C) plus one additional cycle at 72°C for 7 min as a final elongation step. The 16S rDNA PCR amplicons were purified with Exonuclease-I and Shrimp Alkaline Phosphatase (Exo-Sap, Fermentas, Life Sciences) following the manufacturer's standard protocol. Sequence analyses of the purified DNAs were performed using a Big Dye Terminator cycle sequencing kit V3.1 (Applied Biosystems) and an Applied Biosystems 3130XL Capillary DNA Sequencer machine. Sequence similarities were found by BLAST analysis [39] using the GenBank DNA databases (http://www.ncbi.nlm.nih.gov/) and the Ribosomal Database Project (RDP). Phylogenetic analyses of the 16S rRNA gene sequences were conducted with Molecular Evolutionary Genetics Analysis (MEGA) software, version 5 [40]. Trees were constructed by using neighbor-joining method [41]. The sequence was deposited in GenBank database under the accession number KC959984.

### 2.4. Degradation of PCP by Isolated Strain

The kinetics of the PCP removal under different conditions were conducted in 500 mL flasks, sealed with cotton stoppers, containing 100 mL of mineral salt medium (MSM) adjusted to pH 6.9, supplemented with 1% glucose and inoculated with 1% of 10^6^ CFU/mL of the strain FAS23. The MSM contained the following components at the specified concentrations (in g/L): KH_2_PO_4_, 0.8; Na_2_HP_4_, 0.8; MgSO_4_ *·*7H_2_O, 0.2; CaCl_2_ *·*2H_2_O, 0.01; NH_4_Cl, 0.5, plus 1 mL of trace metal solution which includes (in mg/L) FeSO_4_ *·*7H_2_O, 5; ZnSO_4_ *·*7H_2_O, 4; MnSO_4_ *·*4H_2_O, 0.2; NiCl*·*6H_2_O, 0.1; H_3_BO_3_, 0.15; CoCl_2_ *·*6H_2_O, 0.5; ZnCl_2_ 0.25; and EDTA, 2.5. PCP was added to the medium after autoclaving [19]. When necessary, solid MSM plates were prepared by adding 15 g/L bacteriological grade agar. The inoculum was prepared as follows: overnight culture was centrifuged and the pellet was rinsed twice with fresh MSM. PCP removal was monitored during 144 h of incubation by varying different parameters: (i) initial pH; (ii) initial PCP concentrations: 20, 50, 100, 200, and 300 mg/L corresponding to 0.075 mM, 0.19 mM, 0.37, 0.75 mM, and 1.14 mM, respectively; (iii) temperature of incubation: 25, 30, and 37°C; (iv) NaCl concentrations: 10 g/L, 30 g/L, 60 g/L, and 100 g/L; (v) the addition of nonionic surfactant Tween 80 (40 mg/L). Bacterial cell growth was evaluated by measuring the optical density at 600 nm using UV-VIS spectrophotometer (Spectro UVS-2700 Dual Beam Labomed, Inc) every 24 h of the incubation. Three controls were used: PCP-free MSM, uninoculated PCP containing MSM, and PCP containing MSM inoculated with heated inactivated cells. The cell suspension was centrifuged (5 min, 8000 rpm) and the supernatant was filtered through 0.22 *μ*m filters [16]. Samples of 100 *μ*L were applied to C18 reverse phase column (LiChrospher 100 RP-18 endcapped column, 250 mm × 4.6 mm i.d., and particle size of 5 *μ*m) at a flow rate of 1 mL min^−1^. The retained molecules were eluted over 35 min using the following gradient: 1% (v/v) phosphoric acid in water for 4 min, followed by an increase to 100% (v/v) acetonitrile within 21 min which was kept constant for 5 min and then decreased back to initial concentration and kept constant for another 5 min. PCP was quantified using external standards method. Percent removal was estimated using the following formula: removal (%) = area − area/area [42].

### 2.5. Statistical Analysis

Data were subjected to analysis of variance using SPSS software (version 14.0) and the mean differences were compared by Student-Newman-Keuls comparison test. A *P* value of less than 0.05 was considered statistically significant (test at *P* < 0.05). Three replicates were prepared for each treatment.

## 3. Results

### 3.1. Isolation, Identification of FAS23 Strain, and 16S rDNA Sequence Based Phylogenetic Analysis

**Figure 1:**
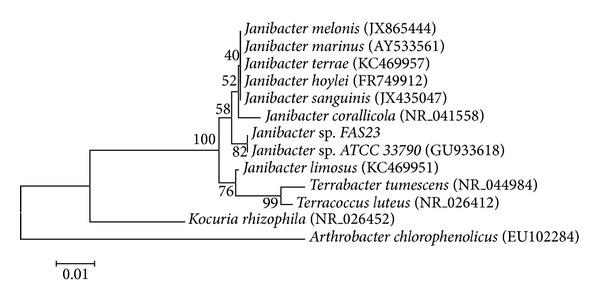
The phylogenetic position of* Janibacter* sp. strain in relation to some members of actinobacteria (genus of* Janibacter*,* Sphingomonas, Terrabacter, Terracoccus, Kocuria,* and* Arthrobacter*) based on 16S rRNA gene. Bootstrap values for a total of 1000 replicates are shown at the nodes of the tree. The scale bar corresponds to 0.05 units of the number of base substitutions per site changes per nucleotide.

The bacterial strain FAS23 was isolated from the saline and arid sediment. The morphological aspect of FAS23 strain culture on the isolation medium YEM showed opaque, pale, cream, and convex colonies with glistening surface. Cells were Gram-positive, rod-shaped, and positive for catalase and oxidase tests. No growth under anaerobic conditions and no spore formation were recorded. The optimal growth conditions of FAS23 strain were pH of 7.0–8.5 and a temperature range of 28–30°C. The strain was able to grow at a range of salt concentrations from 1 to 100 g/L of NaCl. 16S rDNA sequencing and phylogenetic analysis allowed the assignment of FAS23 strain to* Janibacter* sp. (Figure 1).

### 3.2. The Optimum Growth Conditions of* Janibacter* sp. Strain

The effect of physiological and biochemical variations (glucose supplement, temperature, pH, PCP concentration, and presence of biosurfactant) on bacterial growth of* Janibacter* sp. FAS23 and PCP removal was studied.

#### 3.2.1. Effect of Glucose on the Growth of* Janibacter* sp. and PCP Removal

**Figure 2:**
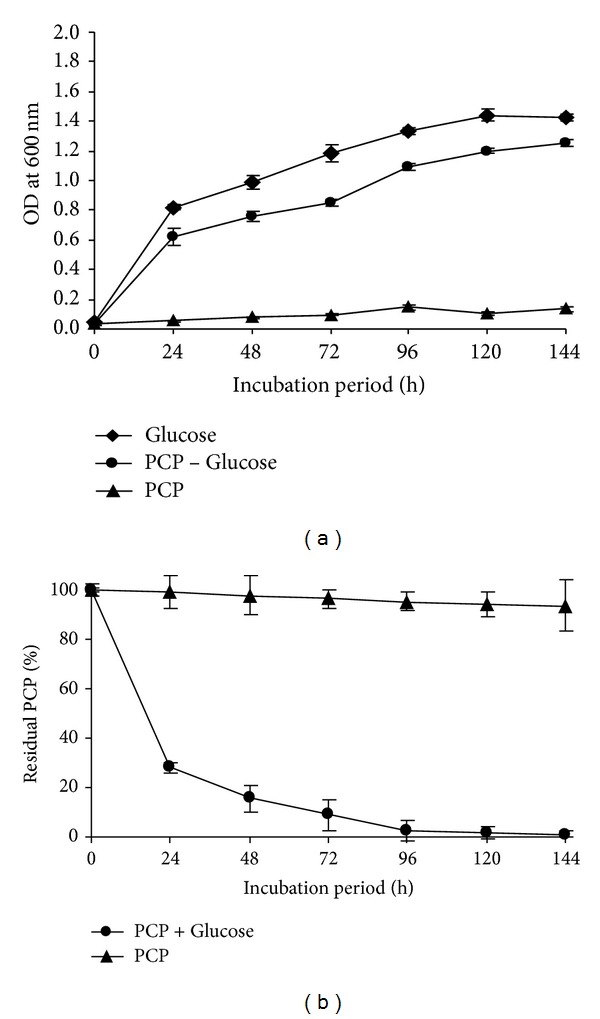
The growth (a) and the PCP removal (b) in the presence and in deficiency of the supplementary carbon source (glucose: 1%) by* Janibacter* sp. FAS23. Error bars represent the standard deviation.

The effect of glucose as cosubstrate on the growth of* Janibacter* sp. strain and PCP removal was studied in MSM. The result showed that the growth of the strain was possible only after the addition of glucose (Figure 2(a)). As well, the PCP was efficiently removed in the presence of glucose, and 71.84% of PCP was degraded within 24 hours and more than 90% after 72 h (Figure 2(b)). The obtained results indicated the phenomenon of cometabolism in which microorganisms do not obtain energy from the transformation reaction; they rather require another substrate for growth [43].

#### 3.2.2. Effect of pH and Temperature on the Growth of the Strain and PCP Removal

**Figure 3:**
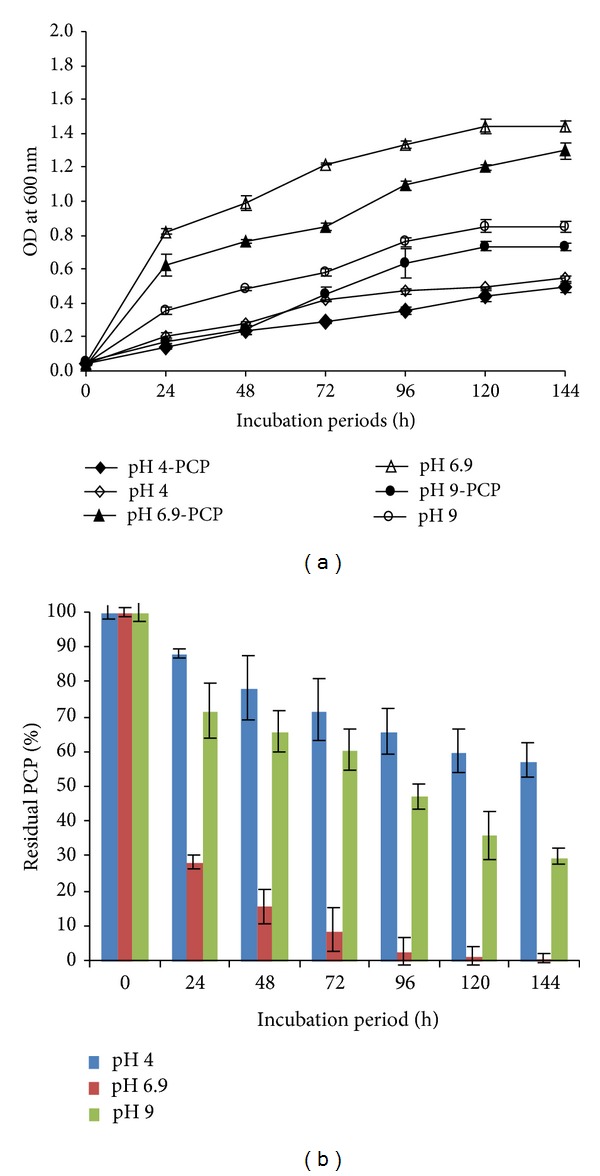
(a) Growth of* Janibacter* sp. at different pH of culture medium: pH 4.0, pH 6.9, and pH 9.0 with 20 mg/L of PCP at 30°C. (b) Effect of different pH on the PCP removal efficiency by* Janibacter* sp. Error bars represent the standard deviation.

**Figure 4:**
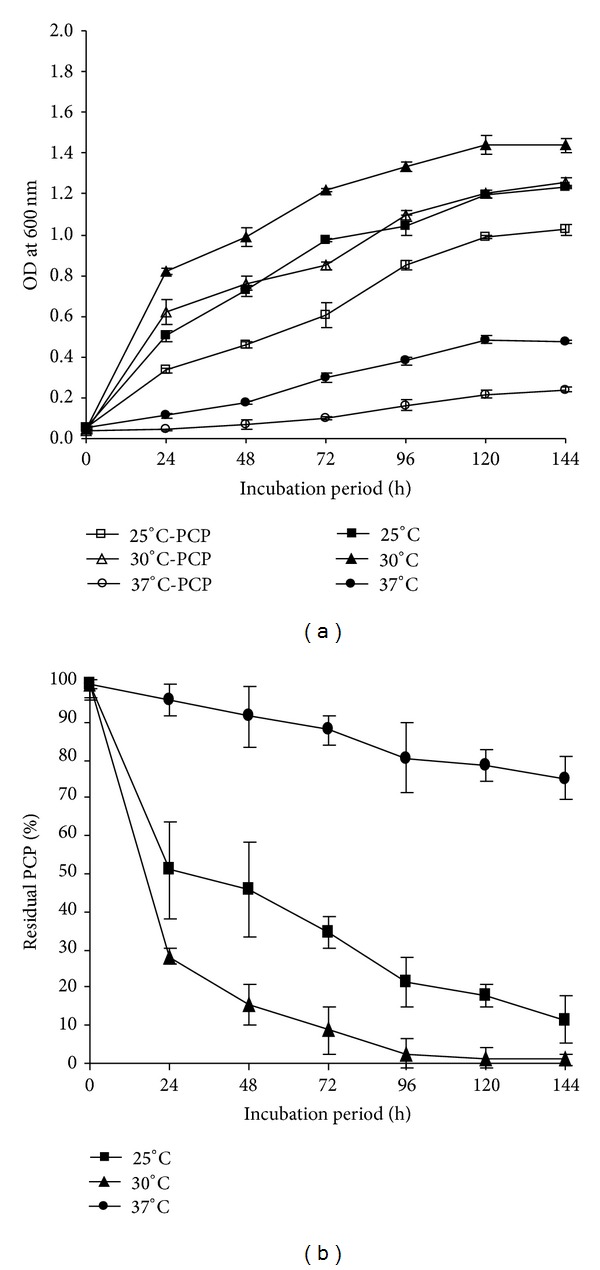
(a) Growth of* Janibacter* sp. at different temperatures in presence of 20 mg/L of PCP and at pH 6.9. (b) Effect of temperature changes on the PCP removal efficiency by* Janibacter* sp. Error bars represent the standard deviation.

The effect of pH variations (4.0, 6.9, and 9.0) on the growth and PCP removal was assessed (Figure 3). At both pH 4.0 and 9.0, a low rate of growth and PCP removal was observed after 24 and 48 h of incubation. However, after 144 h of incubation, the rate of PCP removal has reached values of 44.80% and 70.22% at pH 4.0 and pH 9.0, respectively. The optimum growth and PCP removal were however observed at pH 6.9, as we noted a significant removal of PCP of 71.84%, 84.47%, and 99.06% after 24, 48, and 144 h of incubation, respectively. The strain FAS23 was able to grow in the temperature range of 25–37°C, with an optimum at 30°C. At 25 and 37°C, the growth of the bacterial strain, as well as PCP removal, was affected (Figure 4). However, at 25°C, the strain showed a better growth and PCP removal compared to temperature of 37°C. Likewise, the PCP removal was optimal at 30°C reaching 71.84% and 99.06% after 24 h and 144 h of incubation, respectively.

#### 3.2.3. Effect of PCP Amount on the Growth and PCP Removal by* Janibacter*

**Figure 5:**
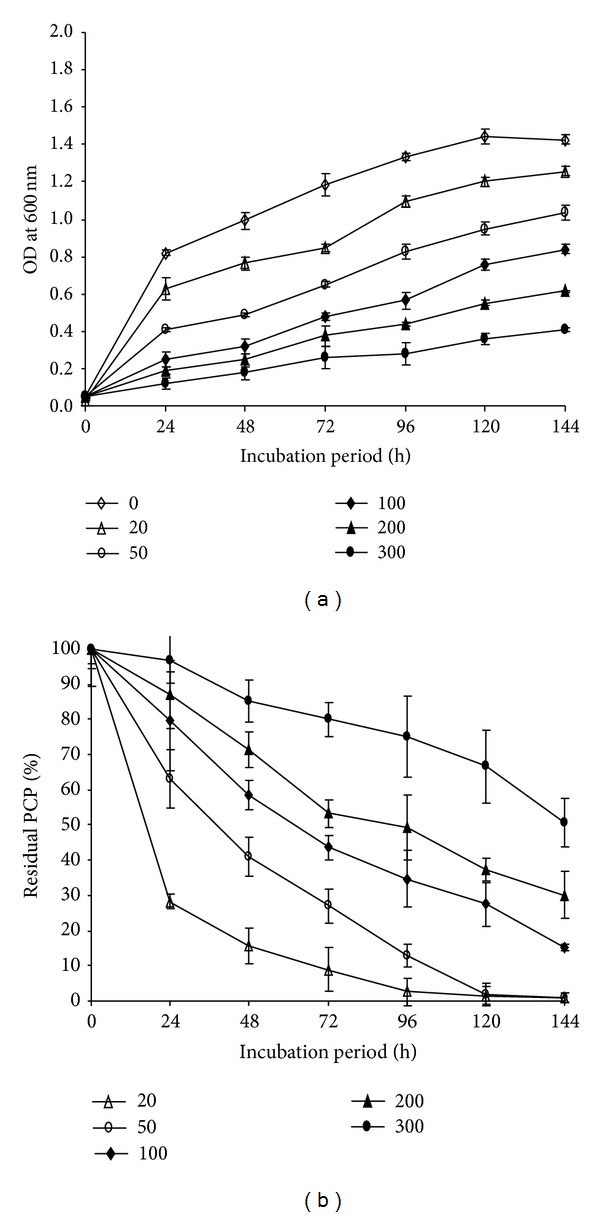
(a) Growth of* Janibacter* sp. in the presence of 0, 20, 50, 100, 200, and 300 mg/L of PCP. (b) Effect of different PCP concentrations on the PCP removal by* Janibacter* sp. Error bars represent the standard deviation.

Variation of PCP amount in the medium showed that the growth of the strain, as well as PCP removal, decreased with the increase of PCP concentration (Figure 5). At low concentrations (20 and 50 mg/L), the bacterial strain was able to remove the majority of PCP after 72 h of incubation time. Up to 100 mg/L, 50% of PCP could be removed after 72 h of incubation. However, with higher concentrations (200 and 300 mg/L), equivalent level of PCP removal could be reached if the incubation time is extended.

#### 3.2.4. Effect of Various NaCl Concentrations on the PCP Removal

**Figure 6:**
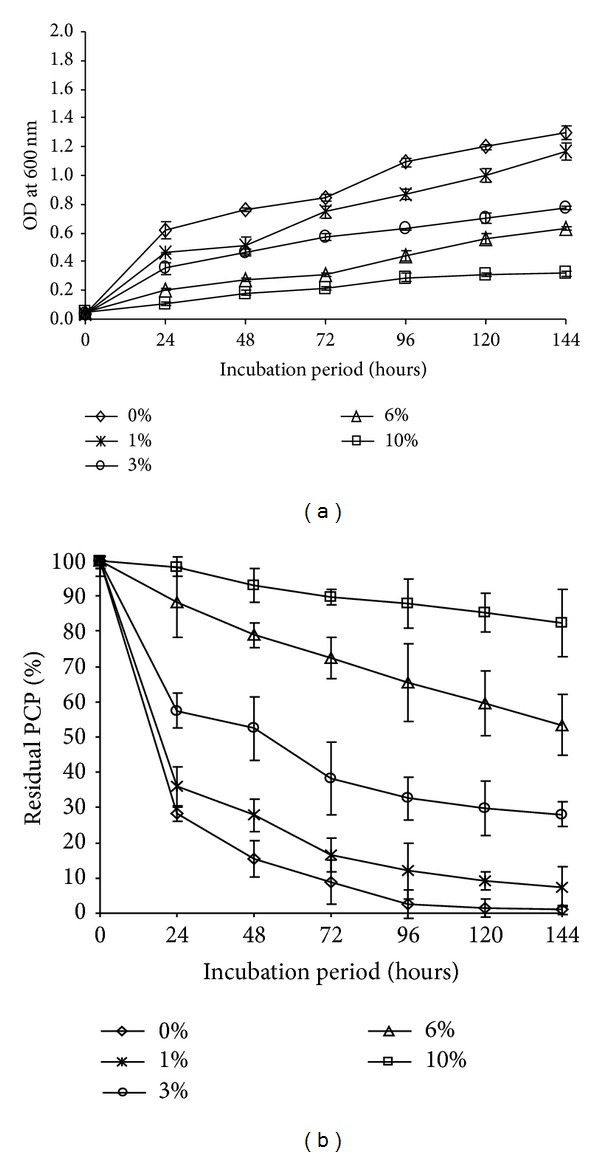
(a) Growth of* Janibacter* sp. in MS medium supplemented with different concentrations of NaCl: 0%, 1%, 3%, 6%, and 10% in the presence of 20 mg/L of PCP at 30°C. (b) Effect of NaCl on the PCP removal efficiency by* Janibacter* sp. Error bars represent the standard deviation.

The strain was tested for its ability to remove PCP (20 mg/L) at different NaCl concentrations (0%, 1%, 3%, 6%, and 10%). The best rate of growth and PCP removal was recorded at 1% of NaCl (more than 92% after 144 h of incubation). The growth and thus the capacity of PCP removal were inhibited when the concentration of sodium chloride was increased (Figure 6). At 3% NaCl, the PCP removal was 72% after 144 h of incubation. When the NaCl concentration was increased to 6% and 10%, PCP removal falls to 46.53% and 17.62%, respectively.

#### 3.2.5. Effect of Nonionic Surfactant Tween 80 on the Biodegradation of PCP

**Figure 7:**
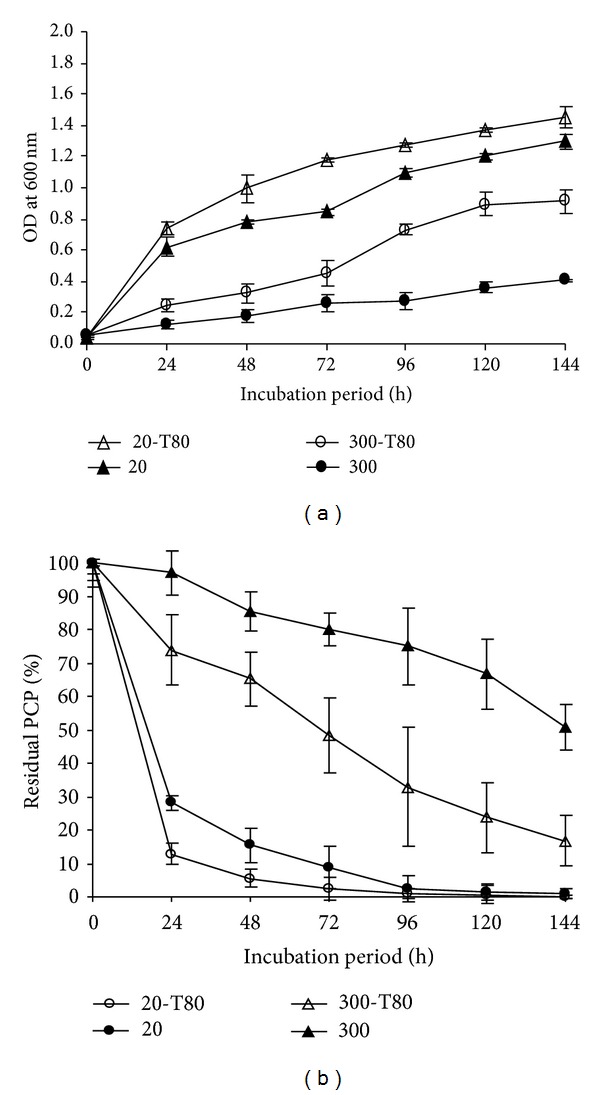
(a) Growth of* Janibacter* sp. in MS medium supplemented with nonionic surfactant Tween 80 (40 mg/L) containing 20 and 300 mg/L of PCP. (b) Effect of nonionic surfactant Tween 80 on the PCP removal efficiency by* Janibacter* sp. Error bars represent the standard deviation.

In this study, the nonionic surfactant Tween 80 was found to enhance the growth and PCP-biodegradation process (Figure 7). Interestingly, removal of high amount of PCP (300 mg/mL) was improved by 30% after 72 h of incubation, compared to the control (Figure 7(b)).

## 4. Discussion

The strain FAS23 isolated from saline sediment collected from Tunisian arid ecosystems was identified as an actinobacterium belonging to the genus* Janibacter* sp., with respect to morphological and biochemical tests and 16S rRNA gene sequence. Despite their known high potential in recalcitrant compounds biodegradation [29, 33], bacteria of the genus* Janibacter*, described in this study, are reported for the first time for their ability to degrade PCP. In biodegradation process, glucose is commonly used as an additional source of carbon and energy and is the most metabolizable sugar which supported a maximum growth [44]. In this context, our results are in agreement with those of Singh et al. [45] and Singh et al. [15] who reported the enhancement of bacterial growth and PCP-degradation process using MSM supplemented with 1% of glucose [43]. This effect can be explained by the connection of the two substrates metabolism. In fact, the NADH provided by glucose metabolism may increase the biomass and thus increase the total activity for PCP metabolizing [46].

In the present study, PCP removal is affected by pH variation. As it was reported by Premalatha and Rajakumar [43], Wolski et al. [47], Barbeau et al. [48], and Edgehill [22] for* Arthrobacter* and different* Pseudomonas* species, the neutral pH was found to be optimal for PCP degradation. However, for other bacterial species, such as* Sphingomonas chlorophenolica*, PCP degradation was more important at pH 9.2 [49].

Temperature is another important environmental factor that can influence the rate of pollutants degradation [48]. The optimal temperature for the PCP removal was recorded at 30°C, but lower temperatures (25°C) allowed significant removal than the upper values. These results were in accordance with those of Wittmann et al. [9] and Crawford and Mohn [50]. Overall, deviation in pH and temperature from the optimum results in alteration of microbial growth and metabolism, as well as the pollutants properties [51, 52].

The effect of different concentrations of PCP on growth of the strain proved that the PCP removal was more efficient at low concentrations (20 mg/L). This result was coherent with data of Webb et al. [53] reporting that all strains tested were able to degrade up to 90% of the PCP, when the concentration was 10 mg/L. Moreover, as it was revealed by Karn et al. [16], the ability of PCP removal of* Janibacter* sp. decreases when PCP concentration was increased. Furthermore, we found that the removal ability by* Janibacter* sp. has reached 40% after 144 h of incubation, when PCP concentration of 300 mg/L was used. These results are in accordance with those of Chandra et al. [54] for* Bacillus cereus* strain and may suggest that these bacteria may tolerate and remove high concentrations of PCP if we increase the incubation time. On the contrary, Kao et al. [55] reported that no PCP removal was detected with PCP concentrations of 320 mg/L even after 20 days of incubation.

As for bioremediation, the strain should possess not only the high removal efficiency for the target compounds but also the strong abilities of adapting some conditions such as pH, temperature, and salinity fluctuations. In this study, it was shown that* Janibacter* sp. was able to remove PCP even with salinity fluctuations (less than 10%). These results were in accordance with those of Gayathri and Vasudevan [56] suggesting that the reduction in phenolic components removal efficiency above 10% NaCl may be due to increase in salinity. These results indicated that* Janibacter* sp. strain has an inherent flexibility to adapt to salinity fluctuations.

The use of surfactants has the potential to increase the biodegradation rate of hydrophobic organic compounds in contaminated environments. Nonionic surfactants are usually used in the bioavailability studies due to their relatively low toxicity compared to ionic surfactants [57]. The enhanced biodegradation in the micelles solution can be attributable to the increased solubility, dissolution, and bioavailability of compound to bacteria [58] and the surfactant enhanced substrate transport through the microbial cell wall [59]. The effects of the surfactants on PCP removal have been invariably attributed to the increased solubility and dissolution of PCP or enhancement of mass transport in the presence of surfactants. In this context, at high concentration of PCP (300 mg/L), Tween 80 increases the removal rate of PCP when the Tween 80 concentration is 40 mg/L. The enhancement of PCP removal was slightly detected when the concentration of PCP is 20 mg/L. These results can be confirmed with the study of Cort et al. [60] when the biodegradation rate of PCP was enhanced for the concentration of PCP at 140 and 220 mg/L but it was inhibited for the concentration of PCP at 100 and 50 mg/L. Consequently, successful integration of PCP and Tween 80 degradation was achieved by* Janibacter* sp. strain.

## 5. Conclusion

In this study, a novel efficient PCP-degrading actinobacterium (*Janibacter* sp.) was isolated from saline soil of arid land and investigated for its physiological characteristics.* Janibacter* was able to remove high concentration of PCP and to tolerate fluctuation of NaCl. This removal potential was moreover accelerated by the addition of Tween 80. This study suggested that strain* Janibacter* sp. could be widely used for PCP bioremediation of polluted arid/extreme environments.
